# Concurrent Infection With the Filarial Helminth *Litomosoides sigmodontis* Attenuates or Worsens Influenza A Virus Pathogenesis in a Stage-Dependent Manner

**DOI:** 10.3389/fimmu.2021.819560

**Published:** 2022-01-24

**Authors:** Gareth R. Hardisty, Johanna A. Knipper, Alison Fulton, John Hopkins, Bernadette M. Dutia, Matthew D. Taylor

**Affiliations:** ^1^ The Roslin Institute, University of Edinburgh, Edinburgh, United Kingdom; ^2^ Institute of Immunology and Infection Research, Ashworth Laboratories, University of Edinburgh, Edinburgh, United Kingdom

**Keywords:** helminth, coinfection, mouse, respiratory virus, filariasis, influenza A virus

## Abstract

Filarial helminths infect approximately 120 million people worldwide initiating a type 2 immune response in the host. Influenza A viruses stimulate a virulent type 1 pro-inflammatory immune response that in some individuals can cause uncontrolled immunopathology and fatality. Although coinfection with filariasis and influenza is a common occurrence, the impact of filarial infection on respiratory viral infection is unknown. The aim of this study was to determine the impact of pre-existing filarial infection on concurrent infection with influenza A virus. A murine model of co-infection was established using the filarial helminth *Litomosoides sigmodontis* and the H1N1 (A/WSN/33) influenza A virus (IAV). Co-infection was performed at 3 different stages of *L. sigmodontis* infection (larval, juvenile adult, and patency), and the impact of co-infection was determined by IAV induced weight loss and clinical signs, quantification of viral titres, and helminth counts. Significant alterations of IAV pathogenesis, dependent upon stage of infection, was observed on co-infection with *L. sigmodontis*. Larval stage *L. sigmodontis* infection alleviated clinical signs of IAV co-infection, whilst more established juvenile adult infection also significantly delayed weight loss. Viral titres remained unaltered at either infection stage. In contrast, patent *L. sigmdodontis* infection led to a reversal of age-related resistance to IAV infection, significantly increasing weight loss and clinical signs of infection as well as increasing IAV titre. These data demonstrate that the progression of influenza infection can be ameliorated or worsened by pre-existing filarial infection, with the outcome dependent upon the stage of filarial infection.

## Introduction

Filarial helminths infect approximately 120 million people worldwide, and remain commonplace in many low and middle income nations despite the existence of effective treatments and detection methods (1). The filarial helminths *Wuchereria bancrofti*, *Brugia malayi* and *Brugia timori* are referred to as lymphatic filariasis and are a significant global health concern (1). It is common for humans to be infected with multiple microbes at any given time, including commensal organisms and chronic or persistent infections. The incidence of concurrent infection with filarial helminths is highly prevalent (2), and therefore it is important to understand their impact on other infections.

Filarial helminths predominantly stimulate Type 2 immune responses in their host, although mixed Type 1 and 2 responses can develop (3). As with other helminth infections, filarial parasites secrete immunosuppressive molecules that impair host immunity in order to maintain a persistent infection (4), and combined with this, the host downregulates its immune responses during chronic infection to avoid severe disease (5). Thus, hosts tend to develop regulatory and modified Type 2 response during chronicity (6, 7). This development of regulatory and Type 2 immunity during chronic filariasis and other helminth infections can influence systemic immunity, including immune responses to third-party antigens such as allergens and concurrent infections (8, 9).

*Litomosoides sigmodontis* infection of inbred mice is used as a model of human lymphatic filariasis (10), and provides the opportunity to test the impact of filariasis on coinfection. *L. sigmodontis* infection initially stimulates a Type 2 immune response during the larval and juvenile adult stages. However, similar to human infections, it develops a mixed Type 1 and 2 response as the adult parasites become fully mature and release the transmission stage microfilaria (Mf) into the blood stream, at which point the infection is referred to as patent. *L. sigmodontis* coinfection has been shown to both protect against and worsen malaria (11–14), protect against *Leishmania major* (15), increase the severity of LPS-mediated inflammation (16), but has no apparent effect on *Mycobacterium tuberculosis* infection (17).

Influenza A virus (IAV) infections cause virulent pro-inflammatory immune responses (18) hallmarked by ‘type I’ immunity, interferon production and generation of pro-inflammatory cytokines such as IL-6, TNF-α and IL-1β. These, can lead to extensive airway pathology within days of infection, increasing the severity of disease and incidence of mortality (19–21). Whilst *L. sigmodontis* can suppress vaccine-induced antibody responses to IAV (22), the impact of filarial co-infection on viral infections, including acute IAV infection, is unknown. We therefore tested whether infection with *L. sigmodontis*, which resides in the pleural cavity of mice, could affect an acute respiratory challenge with IAV. In particular, we tested the hypothesis that the immune regulatory pathways associated with *L. sigmodontis* infection would protect against IAV-induced pathology, and that the strongest protection would be seen during patent *L. sigmodontis* infection.

## Materials and Methods

### Ethics Statement

Experiments were in undertaken accordance with the United Kingdom Animals (Scientific Procedures) Act of 1986 (PPL 60/4479), and approved by the University of Edinburgh Animal Welfare and Ethical Review Body.

### Animals and Infections

Female BALB/c mice were purchased from Charles River and maintained under specific pathogen free conditions at the University of Edinburgh. Mice were used at 6–8 weeks of age. To maintain the *L. sigmodontis* lifecycle, *L. sigmodontis* infected jirds (*Meriones unguiculatus*) were used to infect haematophagous tick parasites (*Ornithonyssus bacoti*) in order to generate L3 stage larvae (23). Mice were infected s.c. on the upper back with 30 *L. sigmodontis* L3 stage larvae. *L. sigmodontis* larvae or adults were recovered from the pleural cavity by lavage and counted using a dissection microscope. To quantify blood Mf, 30 μL of tail blood was collected in FACS lysing solution (Becton-Dickinson) and the Mf counted using a haemocytometer. IAV infections were performed intranasally with 5x10^3^ PFU A/WSN/33, a mouse H1N1 influenza A strain (Dr D. Jackson, University of St Andrews, St Andrews, UK), either 12, 34 or 68 days (d) post *L. sigmodontis* or mock infection. A/WSN/33 was grown in MDCK cells as described previously (24). Mice were weighed daily and assessed for visual signs of clinical disease as described previously (25, 26). Briefly, signs of infection were scored as follows, reduced mobility/activity (0-3), ruffled fur/piloerection (0-3), hunched posture (0-3) and increased or laboured breathing (0-3). The severity score presented is a sum of these criteria.

### Influenza Viral Plaque Assay

MDCK cells were grown to confluence in 6 well plates (Corning) in DMEM (Gibco) containing 5% heat inactivated foetal calf serum (Gibco), 1% Penicillin and Streptomycin (Gibco) and 1% L-glutamine (Gibco). The left lobe of the lungs was recovered and mechanically homogenised with a TH homogeniser (OMNI) in 1.5 ml serum free DMEM before supernatants were recovered following centrifugation at 3000 rpm for 5 mins at 4°C. 10-fold serial dilutions of supernatants were placed onto MDCK cells in duplicate for 1 hour at 37°C. After removal and washing with DPBS (Gibco), a layer of 1% agarose containing 1 x EMEM (Invitrogen), 7.5% fraction V BSA (Sigma), 1% L-glutamine, 7.5 NaHCO_2_ (Invitrogen) 1M Hepes (Sigma) 1% Dextran (Sigma) 1% Penicillin and Streptomycin and 2μg/ml N-acetyl trypsin from bovine pancreas type V-S (Sigma) was added, plates were inverted and cultured for 3 days at 37°C, 5% CO_2_. Cells were fixed in 10% neutral buffered formalin (Sigma) and stained with 0.1% toluidine blue O (Sigma) for 20 minutes before plaques were quantified.

### IFN-γ and IL-10 qPCR

A segment of the left lung lobe was homogenised in Trizol reagent (Thermo) in a tissue homogeniser (Precellys) with ceramic beads. RNA was then isolated with phenol/chloroform extraction according to manufacturer’s instructions and quantified with a NanoDrop (Thermo). cDNA first strand synthesis was performed with MultiScribe Reverse Transcriptase (Thermo) in a c1000 touch, thermal cycler (Bio-Rad). Murine 18s, IFN-γ and IL-10 were detected with SsoAdvanced Universal SYBR Green Supermix (Bio-Rad) on a StepOnePlus Real-Time PCR System (Thermo). Results are shown as 2^-(ΔΔCT) values.

### Statistical Tests

Weight loss data were analysed by General Linear Model and Tukey’s method for pairwise comparisons in Minitab 20 (Minitab LLC). Clinical severity rank scoring data was analysed by Mann-Whitney non-parametric analysis in Prism 9 (Graphpad). Influenza viral titre was analysed by parametric, two tailed, unpaired T test in Prism 9. QPCR data for IL-10 and IFN-γ mRNA was analysed in JMP by two-way analysis of variance and Tukey’s method for pairwise comparisons.

## Results

### Larval-Stage *L. sigmodontis* Infection Reduces the Severity of Influenza A Clinical Signs

**Figure 1:**
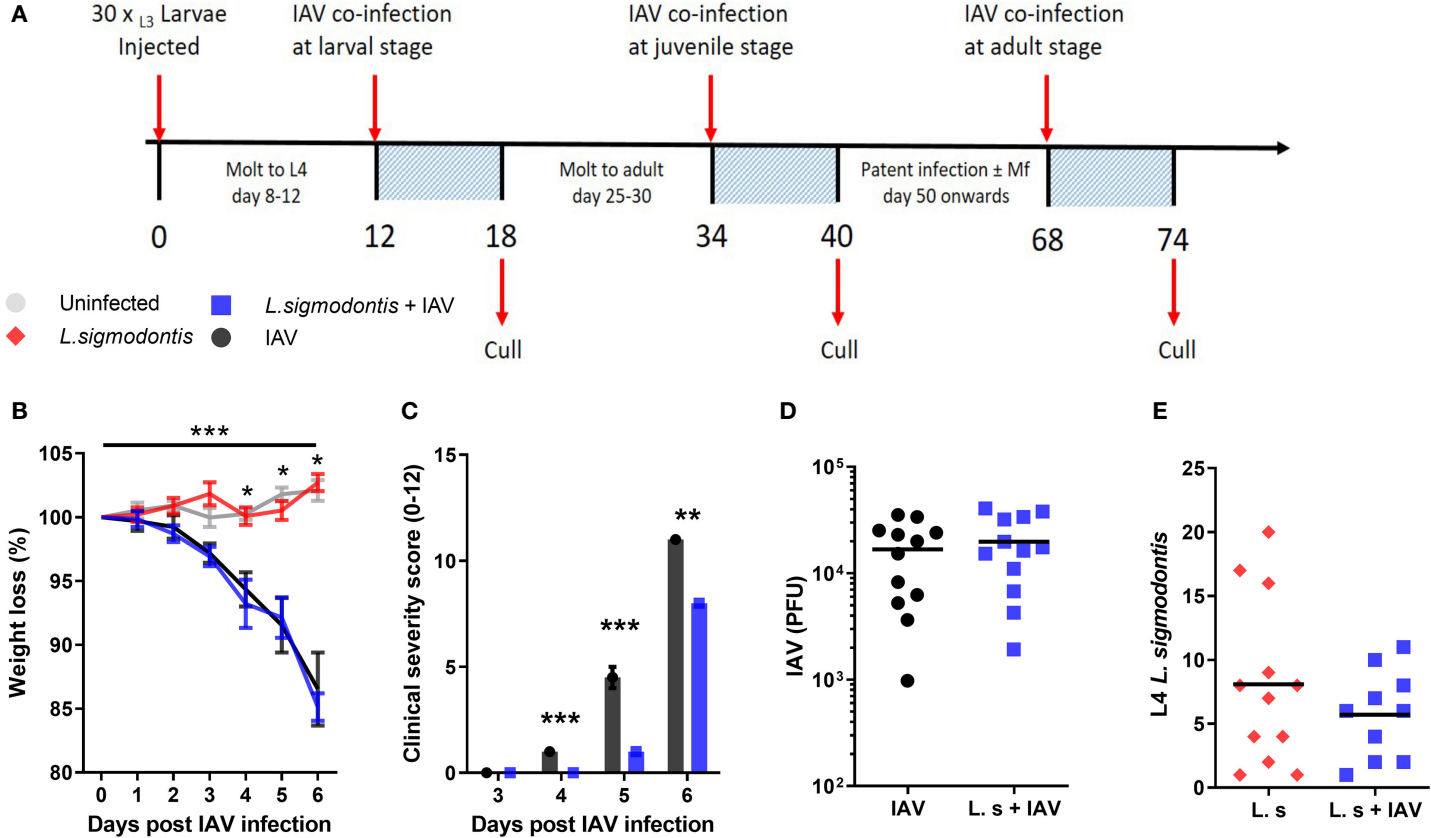
Larval stage *L. sigmodontis* infection reduces the severity of influenza A clinical signs. **(A)** Co-infection timeline. Mice were infected with 30 L3 *L. sigmodontis* larvae subcutaneously. 5x10^3^ PFU IAV was then given intranasally at either d 12 (larval stage), 34 (juvenile adult stage) or 68 (patent infection) pi. Mice were culled 6 d after IAV infection. **(B)** Weight loss over time (d post IAV infection). Mean ± SEM shown. ***Significant effect between groups dependent upon time point, p < 0.001 (GLM). *p < 0.05 between IAV single infection and uninfected group, and co-infected and uninfected groups (Tukey’s HSD). **(C)** Combined IAV clinical severity score. Median ± MAD shown ***p < 0.001, **p < 0.01 (Mann Witney U test). **(D)** IAV titre (PFU) in lung homogenates 6 d post IAV infection. Mean and individual mice shown. **(E)** Numbers of *L. sigmodontis* (L4 larvae) recovered in pleural cavity lavage 6 d post IAV infection. Mean and individual mice shown. All panels show combined data from two independent experiments, (n=12 for all groups).

We first tested whether early larval stage *L. sigmodontis* infection could alter the progression of an acute IAV infection in the lung. Thirty L3 stage *L sigmodontis* larvae were given by subcutaneous injection into the back of 8-week-old female BALB/c mice to mimic the physiological route of infection. *L. sigmodontis* L3 larvae migrate to the pleural cavity *via* the lymphatics over the first 3-4 days of infection. They moult to the fourth larval (L4) stage between 8 -12 (d) post infection (pi) (27), and then to the adult stage between 25 – 30 d pi. Mice were challenged with 3x10^6^ PFU influenza A infection intranasally, or mock infected, on d 12 of *L. sigmodontis* infection so that the 6-day course of influenza infection would take place during the L4 stage (**Figure 1A**), which is associated with a low-level Type 2 immune response (28, 29).

Weight loss was monitored over the 6-d course of co-infection as a clinical sign of IAV severity. This was not significantly altered in mice co-infected with larval stage *L. sigmodontis* compared with mice infected with IAV alone (**Figure 1B**). In contrast, other clinical signs associated with IAV infection including reduced movement, hunching and piloerection were significantly reduced in mice co-infected with larval stage *L. sigmodontis* (**Figure 1C**). At d 5 post IAV infection the average clinical score in IAV infected mice was 5.2 ± 0.75 while co-infected mice were 1.0 ± 0.45, with some mice not yet showing clinical signs and thereby scored as 0. To determine whether the reduced clinical signs correlated with lower viral replication, we measured the IAV lung titre 6 d post-IAV infection. IAV lung titres were not significantly altered in co-infected mice at 6 d post IAV infection (**Figure 1D**). There was also no difference in the number of *L. sigmodontis* L4 larvae recovered from co-infected mice and those infected with *L. sigmodontis* alone (**Figure 1E**).

### Juvenile Adult-Stage *L. sigmodontis* Infection Delays Weight Loss and Progression of Influenza A Clinical Signs

**Figure 2:**
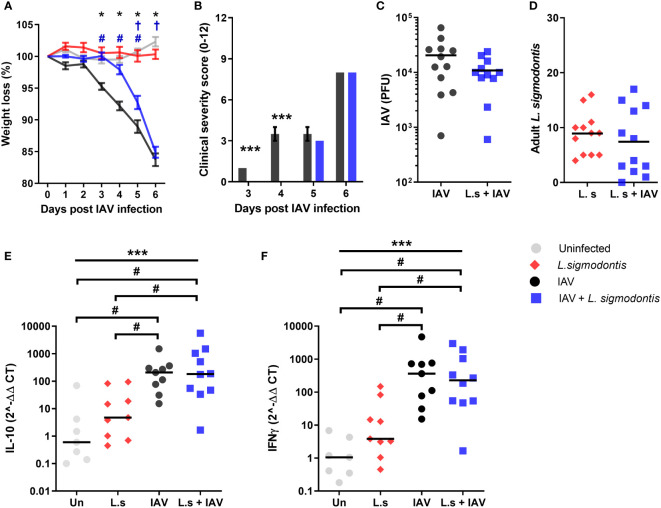
Juvenile adult stage of *L. sigmodontis* infection delays weight loss and progression of influenza A clinical signs. All panels show combined data from two independent experiments, uninfected n=8, IAV n=12, *L. sigmodontis* n=12, IAV + *L. sigmodontis* n=12. **(A)** Weight loss over time (d post IAV infection). Displayed as mean ± SEM. ***significant effect between groups dependent upon time point, p < 0.001 (GLM). *p < 0.05 significant effect between IAV and uninfected group (Tukey’s HSD). ^#^p < 0.05 significant effect between IAV infected and coinfected group (Tukey’s HSD). ^†^p < 0.05 significant effect between uninfected and coinfected group, (Tukey’s HSD). **(B)** Combined clinical severity score. Median ± MAD shown, ***p < 0.001 (Mann Whitney U test). **(C)** IAV titre (PFU) in lung homogenates 6 d post IAV infection. Mean and individual mice shown. **(D)** Numbers of adult *L. sigmodontis* recovered from the pleural cavity 6 d post IAV infection. Median and individual mice shown. **(E, F)** Levels of IL-10 **(E)** and IFN-γ **(F)** mRNA in lung tissue, normalised to 18s RNA expression (n=7-12). Mean and individual mice shown. ***Significant effect between groups (p < 0.001, ANOVA), ^#^p < 0.05 (Tukey’s HSD).

Type 2 and immune regulatory responses increase as *L. sigmodontis* infection progresses (30). Thus, we hypothesised that co-infection with *L. sigmodontis* would have a more profound protective effect on IAV co-infection at later time points. *L. sigmodontis* L4 larvae molt towards the juvenile adult stage around 25 - 30 d pi, becoming reproductively mature adults around 55 d pi when they start producing Mf and infection becomes patent. To test whether the juvenile adult stage has a stronger protective effect on IAV co-infection, mice were co-infected with 5x10^3^ IAV on d 34 of *L. sigmodontis* infection (**Figure 1A**). Weight loss was significantly delayed in the mice co-infected with juvenile *L. sigmodontis* parasites 3-5 d post IAV infection (**Figure 2A**). However, these mice reached the same maximum weight loss 6 d post IAV. In concordance, clinical signs of infection were also significantly reduced in co-infected mice 3 and 4 d post IAV infection, yet reached the same severity at 5 and 6 d post IAV infection (**Figure 2B**). These findings were independent of changes in viral lung titre (**Figure 2C**). Similar to the larval stage of *L. sigmodontis* infection, IAV co-infection had no impact on the number of *L. sigmodontis* parasites recovered (**Figure 2D**).

To determine whether the reduced clinical signs were associated with a change in Type 1 or regulatory cytokines, quantitative PCR was used to measure IFN-γ and IL-10 within the lung. Single IAV infection resulted in significantly increased mRNA expression of *IL-10* and *IFN-γ* compared to the naïve controls (**Figures 2E, F**). However, expression of *IL-10* and *IFN-γ* mRNA was unaltered upon co-infection. *L. sigmodontis* infection alone did not result in increased levels of *IL-10* and *IFN-γ* mRNA within the lung.

### Co-Infection During Patent *L. sigmodontis* Infection Increases Susceptibility to IAV, and Blocks Age-Related Resistance to IAV Clinical Signs

*L. sigmodontis* infection becomes patent at around 55 d pi, at which point the adult parasites are fully mature and are releasing Mf into the blood. Patency associates with a switch from a predominant Th2 response towards a mixed Th2/Th1 immune response, and the development of additional layers of immune regulation (16). To determine whether patent *L. sigmodontis* infection alters susceptibility to IAV, mice were co-infected with IAV at d 68 of *L. sigmodontis* infection (**Figure 1**).

**Figure 3:**
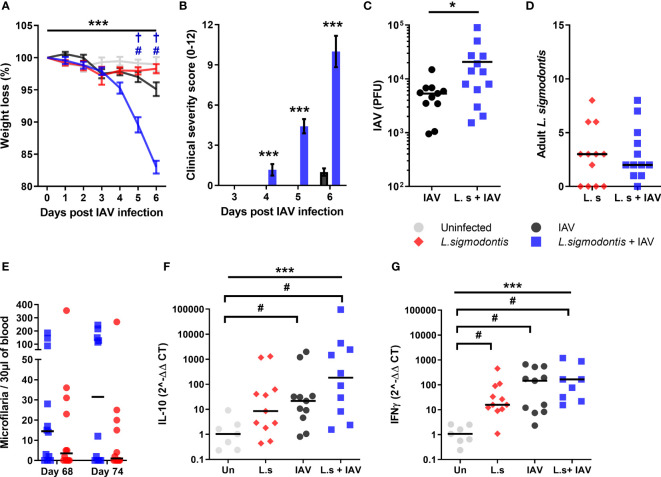
Co-infection during patent *L. sigmodontis* infection increases susceptibility to IAV, and blocks age-related resistance to IAV clinical signs. All panels show combined data from two independent experiments. Uninfected n=12, IAV n=11, *L. sigmodontis* n=12, IAV + *L. sigmodontis* n=13 **(A)** Weight loss over time (d post IAV infection) shown as mean ± SEM, ***significant effect between groups over time, p < 0.001 (GLM). ^#^p < 0.05 significant effect between IAV infected and coinfected group (Tukey’s HSD). ^†^ p < 0.05 significant effect between uninfected and coinfected group (Tukey’s HSD). **(B)** Combined clinical severity score. Median ± MAD shown. ***p < 0.001 (Mann Whitney U test) **(C)** IAV titre (PFU) in lung homogenates 6 d post IAV infection. Mean ± SEM. *p < 0.05 (unpaired T test). **(D)** Numbers of adult *L. sigmodontis* recovered 6 d post IAV infection. Median and individual mice shown (*L.sigmodontis* n=12, IAV + *L. sigmodontis* n=13). **(E)** Mf count in 30 μl blood before and after IAV co-infection. Median and individual mice shown. **(F, G)** Expression of IL-10 **(F)** and IFN-γ **(G)** mRNA in the lung. ***Significant effect between groups, p < 0.001 (ANOVA), ^#^p < 0.05 (Tukey’s HSD).

BALB/c mice show increasing resistance to IAV (A/WSN/33) infection with age (31), and as patent *L. sigmodontis* infection takes over 2 months to develop, the mice were 4-5 months of age at the time of IAV challenge. Consistent with their age, the weight loss due to IAV infection alone was reduced compared to previous time points, with a mean weight loss of 4.9% at 6 d post IAV infection (**Figure 3A**). However, co-infected mice demonstrated significantly higher weight loss in response to IAV infection, with a mean weight loss of 17.0% by d 6. Furthermore, whilst clinical signs of infection were only detected in IAV infected mice at d 6 (average score of 1.0 ± 0.27), co-infected animals demonstrated clinical symptoms by d 4 and progressed to show increasingly severe clinical signs at 6 d post IAV infection with an average score of 10.0 ± 1.17 (**Figure 3B**). Co-infected mice also had a significantly increased IAV lung titre, with a 3.9-fold increase in PFU compared with IAV infection alone (**Figure 3C**).

As with co-infection at earlier stages of *L. sigmodontis* infection, IAV did not alter susceptibility to *L. sigmodontis*. The total numbers of adult *L. sigmodontis* remained similar between co-infected and *L. sigmodontis* infected mice (**Figure 3D**). Co-infection also had no impact on the numbers of circulating Mf in the blood, with *L. sigmodontis* singly and co-infected mice showing similar levels of Mf immediately prior to, and after, IAV infection (**Figure 3E**). Similarly, the incidence of mice developing blood Mf remained the same between groups both before (co-infected 8/14, *L. sigmodontis* only 10/14) and at d 6 of IAV infection (co-infected 9/14, *L. sigmodontis* only 7/14).

Quantitative PCR was used to determine whether the increased lung pathology was associated with alterations in *IFN-γ* or *IL-10* mRNA expression within the lung. There were significant increases in mRNA expression of IL-10 and IFN-γ in the lung as a result of IAV infection compared with uninfected mice, and significantly increased IFN-γ due to *L. sigmodontis* infection alone (**Figures 3F, G**). However, co-infected mice did not display differences in expression of mRNA of either cytokine compared to the single infections.

## Discussion

This study demonstrates that pre-existing *L. sigmodontis* infection can significantly ameliorate or worsen the progression of acute respiratory infection with Influenza A virus (IAV), with the outcome dependent upon the stage of *L. sigmodontis* infection. Co-infection with IAV during the larval and juvenile adult stages of *L. sigmodontis* infection delayed the onset of clinical signs, whilst co-infection during patent *L. sigmodontis* infection increased IAV clinical signs, weight loss, and viral loads.

Mice infected with the intestinal parasite *Trichinella spiralis* at an early enteric stage were found to undergo faster recovery from IAV induced weight loss (32). Infection with pre-patent *L. sigmodontis* also significantly decreased the severity of IAV co-infection, although the protective effect presented as a reduction in the initial severity of infection. This protection was more pronounced during the juvenile adult stage of *L. sigmodontis* infection than the larval L4 stage, with the juvenile adult stage delaying weight loss as well as clinical signs. This contrasts to coinfection with *L. sigmodontis* and Friend retrovirus where L4 stage *L. sigmodontis* infection caused significantly increased splenomegaly and viral loads (33). Decreased IAV severity was independent of viral titre and helminth burden, which were unaltered by co-infection at either of the pre-patent life stages. The dose of IAV was chosen to cause an infection of moderate severity that could modulated up or down by coinfection without mortality. As the initial dose of IAV can instruct early interferon, cytokine and chemokine expression (34), different IAV doses could result in different coinfection outcomes. In particular, *L. sigmodontis* only delayed the onset of clinical signs and weight loss, raising a question of whether the protective effect of *L. sigmodontis* infection would still be sufficient to ameliorate severity during high dose, more pathogenic, IAV co-infection.

IL-10 plays an important regulatory role during *L. sigmodontis* infection (35), and is involved in suppressing cerebral malaria during *L. sigmodontis* co-infection (11). Similarly, concurrent pre-patent *L. sigmodontis* infection suppresses IAV immunisation in an IL-10 dependent manner (22). Administration of the immunomodulatory filarial cystatin (AvCystatin/Av17), which stimulates IL-10 producing Foxp3^+^ Tregs, can also protect against inflammation and weight loss in a model of respiratory syncytial virus inflammation (36). However, at the endpoint, IL-10 expression in the lung homogenates of *L. sigmodontis* and IAV co-infected mice did not correlate with the increased protection. Whilst measuring IL-10 protein production at earlier time points and in specific cell populations would give a more accurate representation of IL-10 activity, this data could suggest an IL-10 independent effect.

In contrast to the protective effect of pre-patent *L. sigmodontis* infection, patent *L. sigmodontis* infection worsened the progression of IAV infection. Mice develop age-related resistance to IAV (31), displaying reduced clinical signs and weight loss as they age. Patent *L. sigmodontis* infection countered this age-related resistance and significantly worsened weight loss and clinical signs of IAV infection, as well as increasing IAV titre. The impaired protection is similar to studies demonstrating that *T. spiralis* infection impairs immunity to murine norovirus (37), but contrasts with *Heligmosomoides polygyrus* and respiratory syncytial virus coinfection where *H. polygyrus* enhanced resistance to the virus through type 1 interferons (38). An increasing number of immune regulatory pathways develop as infection progresses to patency, including Tregs (28, 39), alternatively activated macrophages (40), and T cell-intrinsic dysfunction (10, 29, 41), and increased suppression of third-party immune responses are seen as infection matures (42). An increased immune-regulatory response may have hindered the immune system’s ability to control IAV replication, with the resultant increase in viral burden exacerbating immune pathology. However, as overspill of immune regulation could also protect against pathology, the immune regulatory mechanism would need to inhibit protective, but not pathogenic, immune responses to IAV.

An alternative reason for the increased susceptibility to IAV infection could relate to perturbations in cytokine production during infection. The induction of Type 2 responses by helminths are found to be sufficient to reactivate latent murine γ-herpesvirus infection (43). However, pre-patent *L. sigmodontis* infection stimulates a Type 2 response and protected against influenza signs and weight loss, suggesting that the Type 2 response at patency is unlikely to have worsened the progression of IAV infection. In contrast with pre-patent infection, patent *L. sigmodontis* infection does associate with the development of a mixed Th1/Th2 response (10). Patent *L. sigmodontis* singly infected mice showed increased levels of IFN-γ in their lung homogenates, that were not present during pre-patent infection. IFN-γ, along with IL-5, is part of the protective response against microfilaria (44), and microfilaremia associates with increased severity of LPS-induced inflammation due to elevated IFN-γ, TNF-α, IL-6 and IL-12 expression (16). Similarly, increased IFN-γ correlates with more severe disease in *L. sigmodontis* and *Plasmodium chabaudi chabaudi* co-infected mice. However, the effect of IFN-γ levels on concurrent infections is context dependent. During *Leishmania major* coinfection, *L. sigmodontis*-enhanced IFN-γ responses were associated with a delay in disease onset rather than enhancement of pathology (15), whilst *L. sigmodontis* infection does not affect the generation of Th1 IFN-γ driven responses and susceptibility during concurrent infection with *Mycobacterium tuberculosis* (17). Although *L. sigmodontis* infection did not increase IFN-γ mRNA levels in IAV coinfected mice, IFN-γ is a key factor determining the extent of pathology (26), and future studies should explore IFN-γ as a potential mechanism.

Malaria models have also highlighted the importance of *L. sigmodontis* infection stage on the outcome of coinfection. Similar to IAV coinfection, larval stage *L. sigmodontis* infection protects against pathology in *P. yoelii* and *P. chaboudi* infections. Although it also increases resistance to *P. yoelii*, and co-infection with either malaria species increases resistance to *L. sigmodontis* (12). Patent *L. sigmodontis* infection also worsened pathology in *P. chaboudi* coinfected mice, again mirroring IAV coinfection (13, 14). However, contrasting with IAV and *P. chaboudi*, patent *L. sigmodontis* infection protected 30% of co-infected mice from *P. berghei* infection again indicating that the effects of coinfection are context dependent (34). Not all mice develop patent *L. sigmodontis* infection (defined by Mf circulating in the blood), and the extent of *P. chaboudi* pathology correlated with the presence or absence of blood Mf, with more severe pathology in Mf negative mice (13, 14). Similarly, Mf negative mice showed lower protection against *P. berghei* (13, 14). All but 2 mice developed Mf between d 68 and 74 post-*L. sigmodontis* infection in our study, and so it was not possible to determine whether Mf status impacted the outcome of IAV. As Mf do stimulate IFN-γ production (45), it would be interesting to determine whether the exacerbation of pathology by patent *L. sigmodontis* infection is due to the release of Mf.

This study demonstrates that there are interactions between the filarial helminth and acute respiratory viral infections, and that the presence of a helminth infection can both ameliorate and worsen IAV severity with the outcome dependent upon the stage of helminth infection. Further research is required to elucidate the mechanisms by which this interaction occurs. This suggests that consideration of concomitant infection with filarial helminths may be a significant factor in the treatment and outcome of IAV and other respiratory infections such as SARS-CoV-2, where the expression of type 2 cytokine responses is associated with increased disease severity (46).

## Data Availability Statement

The original contributions presented in the study are included in the article/supplementary material. Further inquiries can be directed to the corresponding author.

## Ethics Statement

The animal study was reviewed and approved by University of Edinburgh Animal Welfare and Ethical Review Body.

## Author Contributions

BD, JH, GH, and MT designed the experiments. GH, JK, AF and MT conducted the experiments and collected the data. GH, BD, and MT analysed the data and wrote the manuscript. All authors contributed to the article and approved the submitted version.

## Funding

This project was funded by the Biotechnology and Biological Sciences Research Council (BBSRC) Institute Strategic Program Grant BB/J004324/1 to The Roslin Institute. GH was funded by a BBSRC Doctoral Training Grant to the Centre for Infectious Diseases, University of Edinburgh. MT, AF, and JK were funded by the Medical Research Council (MRC) UK grant number MR/K020196/1, and the Wellcome Trust grant number 095831. Open access publication costs were provided by the UKRI Open Access Fund. The funders had no role in study design, data collection and analysis, decision to publish, or preparation of the manuscript.

## Conflict of Interest

The authors declare that the research was conducted in the absence of any commercial or financial relationships that could be construed as a potential conflict of interest.

## Publisher’s Note

All claims expressed in this article are solely those of the authors and do not necessarily represent those of their affiliated organizations, or those of the publisher, the editors and the reviewers. Any product that may be evaluated in this article, or claim that may be made by its manufacturer, is not guaranteed or endorsed by the publisher.

## Abbreviations

IAV, Influenza A virus; Mf, microfilaria; L3, third larval stage; d, day; pi, post infection; L4, fourth larval stage.
